# Whole-Genome Shotgun Assembly and Analysis of the Genome of *Streptomyces mobaraensis* DSM 40847, a Strain for Industrial Production of Microbial Transglutaminase

**DOI:** 10.1128/genomeA.00143-13

**Published:** 2013-04-04

**Authors:** Huilin Yang, Tiansheng He, Wenxia Wu, Weitao Zhu, Bai Lu, Wanju Sun

**Affiliations:** Key Laboratory of Functional Small Organic Molecule, Ministry of Education and College of Life Science, Jiangxi Normal University, Nanchang, Jiangxi, China

## Abstract

Here, we report the draft annotated genome sequence of *Streptomyces mobaraensis* strain DSM 40847, which is used in industry to produce microbial transglutaminase. The genome sequence will allow for the characterization of the molecular mechanisms underlying the beneficial properties of this organism.

## GENOME ANNOUNCEMENT

Transglutaminases (TGases) (protein-glutamine γ-glutamyltransferases, EC 2.3.2.13) are a family of enzymes that catalyze an acyl transfer reaction between a free amine group and a γ-carboximide group of protein-bound or peptide-bound glutamine (1). TGases derived from eukaryotes, including human blood coagulation factor XIII (2), human tissue TG (2), pig liver TG (3), and fish liver TG (4), are calcium-dependent, poly-subunit enzymes. To date, there are nine distinct TG isoenzymes in mammals that have been identified at the genomic level, of which eight are structurally and functionally related to the function of TGases, *viz*., TG 1 to 7 and factor XIII (5, 6). In prokaryotes, calcium-independent TGases with only one subunit have been discovered in *Streptomyces* (7) and *Bacillus* (8). Currently, transglutaminases are produced mainly by *Streptomyces mobaraensis* microbial fermentation. Although there are many published papers (9–13) reporting transglutaminase-producing strains of *S. mobaraensis*, not much is known about the genome sequences of these producing strains. We first sequenced the genome of *Streptomyces mobaraensis* DSM 40847, a strain for industrial production of microbial transglutaminase.

The genome was sequenced using the Illumina Solexa Hiseq2000 instrument at Beijing Genomics Institute (BGI) (Shenzhen, China). A library containing 500-bp inserts was constructed. Sequencing was performed with the paired-end strategy of (90, 90)-bp reads to produce 1.2 Gb of filtered sequences, representing an 85.0-fold coverage of the genome. The sequences were assembled into 266 contigs using the Velvet software (14).

Genome annotation was performed at the NCBI Prokaryotic Genomes Automatic Annotation Pipeline. Open reading frames (ORFs) were identified by Glimmer 3.02 (15) and Genemark (16). The resulting translations were used for a BLASTP (17) search against the GenBank NR database, as well as the KEGG (18) and COG (19) databases. tRNA and rRNA genes were identified by tRNAscan-SE (20) and RNAmmer (21), respectively.

The DSM 40847 chromosome is about 7.5 Mbp in length, with an average G+C content of 72.5%. A total of 6,422 protein-coding genes were identified. The genome sequence will represent a valuable shortcut, helping scientists to find genes. The transglutaminase gene was found, and the genes encoding the endogenous proteases TAMEP and TAP, which were used to activate protransglutaminase, were also found. The genome sequence will accelerate the progress of *Streptomyces mobaraensis* research.

### Nucleotide sequence accession numbers. 

This whole-genome shotgun project has been deposited at DDBJ/EMBL/GenBank under the accession number AORZ00000000. The version described in this paper is the first version, AORZ01000000.
